# Twisted-plywood-like tissue formation *in vitro*. Does curvature do the twist?

**DOI:** 10.1093/pnasnexus/pgae121

**Published:** 2024-03-21

**Authors:** Barbara Schamberger, Sebastian Ehrig, Thomas Dechat, Silvia Spitzer, Cécile M Bidan, Peter Fratzl, John W C Dunlop, Andreas Roschger

**Affiliations:** Department of the Chemistry and Physics of Materials, Paris-Lodron University of Salzburg, 5020 Salzburg, Austria; Laboratory of Systems Biology of Gene Regulatory Elements, Berlin Institute for Medical Systems Biology, Max Delbrück Center for Molecular Medicine in the Helmholtz Association, Hannoversche Str. 28, 10115 Berlin, Germany; Ludwig Boltzmann Institute of Osteology of OEGK and AUVA Trauma Centre Meidling, 1st Medical Department, Hanusch Hospital, 1140 Vienna, Austria; Ludwig Boltzmann Institute of Osteology of OEGK and AUVA Trauma Centre Meidling, 1st Medical Department, Hanusch Hospital, 1140 Vienna, Austria; Department of Biomaterials, Max Planck Institute of Colloids and Interfaces, 14476 Potsdam, Germany; Department of Biomaterials, Max Planck Institute of Colloids and Interfaces, 14476 Potsdam, Germany; Department of the Chemistry and Physics of Materials, Paris-Lodron University of Salzburg, 5020 Salzburg, Austria; Department of the Chemistry and Physics of Materials, Paris-Lodron University of Salzburg, 5020 Salzburg, Austria

**Keywords:** curvature, twisted plywood, tissue growth, cell alignment, collagen alignment

## Abstract

Little is known about the contribution of 3D surface geometry to the development of multilayered tissues containing fibrous extracellular matrix components, such as those found in bone. In this study, we elucidate the role of curvature in the formation of chiral, twisted-plywood-like structures. Tissues consisting of murine preosteoblast cells (MC3T3-E1) were grown on 3D scaffolds with constant-mean curvature and negative Gaussian curvature for up to 32 days. Using 3D fluorescence microscopy, the influence of surface curvature on actin stress-fiber alignment and chirality was investigated. To gain mechanistic insights, we did experiments with MC3T3-E1 cells deficient in nuclear A-type lamins or treated with drugs targeting cytoskeleton proteins. We find that wild-type cells form a thick tissue with fibers predominantly aligned along directions of negative curvature, but exhibiting a twist in orientation with respect to older tissues. Fiber orientation is conserved below the tissue surface, thus creating a twisted-plywood-like material. We further show that this alignment pattern strongly depends on the structural components of the cells (A-type lamins, actin, and myosin), showing a role of mechanosensing on tissue organization. Our data indicate the importance of substrate curvature in the formation of 3D tissues and provide insights into the emergence of chirality.

**Significance Statement**

Biological tissues (like compact bones) often consist of multiple fibrous layers that are staggered with a twisting angle relative to each other, thereby improving mechanical performance. The underlying principles of how such tissues are formed and what determines the fiber direction are still debated. In this study, we report the formation of a twisted-plywood-like tissue grown *in vitro* on constant mean and negative Gaussian curvature substrates. We present evidence that for tissue consisting of preosteoblast-like cells, surface curvature is a main determinant for fiber orientation.

## Introduction

Structural biological materials, such as bone, wood, and cuticle, are composite materials (1, 2) and can consist of biopolymers, such as cellulose, chitin, and collagen. These polymers are long and thin, and form fibers or fibrils, which in turn pack parallelly into lamellae (1). Such lamellar structures are ubiquitous in nature, as seen in certain bone types (3), the exoskeleton of crustaceans (4), the cell wall of plants (5), and many more (1). These tissues typically consist of multiple layers of lamellae, giving rise to twisted structures, in which the fiber direction changes from layer to layer.

There are mechanical advantages for a natural material to exhibit tissue twisting. It results in the isotropic material properties of a tissue formed from intrinsically anisotropic building blocks, but more importantly, such layered materials are excellent at resisting the propagation of cracks, which explains the high fracture toughness in so many biological materials (3, 6–9). A well-studied example is lamellar bone found, for example, in osteons of human long bones. There, mineralized fiber bundles of collagen type I build up ∼5-µm-wide lamellae with varying fiber angles to form a twisted material with high toughness (9–13).

Interestingly, in lamellar bone, twisting patterns appear on all length scales (14) and considerably differ between various species: While 3D electron microscopy data from mammalian bone tissue indicate the presence of an unordered phase between highly aligned collagen fiber bundles (15–17), in anosteocytic fish bone, continuous tissue twisting between lamellae is reported (18). Such a structure is reminiscent of twisted plywood, where changing fiber angles between adjacent layers significantly improve the material's mechanical performance. Despite the well-known connection between structure and mechanical function, the processes causing the emergence of twisted plywood structures in nature remain unclear. One theory suggests that self-assembly of these biopolymers into liquid crystalline phases gives rise to these structures (1, 19). Alternatively, it could be alignment of the cells themselves, which cause long-range order. This has been demonstrated in studies of cells seeded on flat fibronectin patches of different shapes (20). Cell alignment then influences extracellular matrix production resulting in the formation of twisted tissue layers. In this study, we explore tissue formation using one in vitro bone-like tissue growth model, where a preosteoblastic cell line (MC3T3-E1) produces tissues containing oriented extracellular matrix material. Although it is difficult to extrapolate our results to other tissue types, it is hoped that an understanding of the processes giving rise to twisted-plywood-like tissues in bone will help us to reach a better understanding of 3D tissue formation in general.

Multilayered tissues are difficult to grow *in vitro* on flat surfaces in 2D, however, for curved surfaces the situation changes, and thick tissues can form in which multiple layers of cells can be found. This is relevant, of course, to processes occurring on the highly curved surfaces found in trabecular (21, 22) and osteonal bone (23), where during bone formation a layer of osteoblasts forms new tissue. The presence of curvature can in turn change the behavior of cells, modulating pattern formation, and influencing the development of helicoidal tissues (24). To understand the development of such tissues, it is thus necessary to determine the role of curvature in the long-range patterning of cells and the extracellular matrix that they produce.

Surface curvature has been shown to be an important geometric signal that plays a role in the fate and behavior of single cells and tissues (25, 26). For instance, mesenchymal stem cells (MSCs) seeded on convex semi-cylinders aligned preferentially along the zero curvature or axial direction. In contrast, MSCs seeded within concave semi-cylinders oriented toward the direction of highest curvature magnitude perpendicular to the cylinder axis (27). MSCs are also known to exhibit “curvotaxis” (28) or curvature-guided migration (27). On negative Gaussian curvature surfaces, they migrate along concave (valley-shaped) directions and on sinusoidal surface cells migrate toward the concave valleys (28, 29). Multicellular tissues formed by murine preosteoblast cells (MC3T3-E1) within prismatic pores show higher growth rates in concave compared with flat regions (30, 31). Tissues grown on triply periodic minimal surfaces (also with negative Gaussian curvature) display higher levels of osteogenic differentiation markers than tissues grown on control scaffolds, indicating that tissues also respond to surface curvature (32). An important hint toward the mechanism controlling this curvature sensing is the observation that a growing tissue has a shape that can be described by the Young–Laplace law (33). This link between pressure (mechanics) and curvature suggests the importance of mechanical signaling in the sensing of curvature by cells and tissues, which is in turn further supported by computational modeling (34, 35). Although cellular mechanosensing clearly plays an important role in curvature-driven cell migration and tissue growth (36, 37), the underlying biological mechanisms are not yet clear. Recent studies suggest the shape and the deformation of the nucleus (38, 39), the amount and arrangement of focal adhesion formation (28, 39), and the organization of the cytoskeleton (39) as potential key players in determining curvature sensing by cells.

An additional aspect that is required to understand how geometry influences the development of multilayered tissues is that cells may display preferential symmetries, preferring to orient with a left- or right-handed twist with respect to their surroundings. The actin cytoskeleton of single cells cultured on a circular patch exhibits a left-right asymmetry (40–42). Such chiral arrangements were also observed in cell monolayers confined to surface patches for several cell types (42–44). Remarkably, it has also been shown that interfering with certain actin-associated proteins or tempering actin polymerization by drugs can reduce, eliminate, or even reverse this effect in single cells and cell monolayers (42). Symmetry breaking in cell and tissue patterning can also be observed on hyperbolic surfaces, where preosteoblast-like cells were shown to self-organize into a left-handed chiral pattern (33). It is hard to pin down the ultimate cause of the asymmetric behavior of a growing tissue, as asymmetries occur across multiple length scales and may be causally related (45, 46). Single-cell experiments revealed that the asymmetry of the cytoskeletal proteins plays an important role in the polarity of cells, which in turn influences the asymmetry of tissues and organs (40–42).

Hence, it is possible that multilayered twisted-plywood-like tissues develop through the complex interaction between mechanical signaling, curvature sensing, and the innate asymmetry of the tissue-producing cells themselves. To search for such interactions, investigation of tissues formed by cells needs to consider the 3D nature of the tissues and especially the effect of curvature on cells, as well as potential underlying cellular and subcellular mechanisms causing the emergence of chiral patterns on higher hierarchical levels. As tissue shape and, thus, surface curvature change during growth, curvature-induced cell, and tissue alignment may adapt with time. We explore the coupling between these aspects by growing thick tissues produced by MC3T3-E1 preosteoblast cells on rotationally symmetric scaffolds of constant mean but negative Gaussian curvatures. In this sense, the term “tissue” refers to all the organic matrix present on the scaffold which may be a combination of cells and extracellular matrix. Tissue orientation is quantified as a function of growth time by evaluating actin stress-fiber and collagen-fiber orientation with respect to the principal curvature directions. Mechanistic insight is gained by comparing wild-type (WT) cells with cells exhibiting impaired nuclear mechanosensitivity (lamin A/C-deficient cells), as well as treatment for inhibited actin-polymerization (Latrunculin A), inhibited myosin II (Blebbistatin), and enhanced actin stress-fiber formation (transforming growth factor-β1 [TGF-β1]). By measuring how cell and tissue orientation evolve with time, we start to unravel the complex mechanisms determining large-scale tissue organization.

## Results

### Emergence of left-handed chirality in tissue grown on negative Gaussian curvature surfaces

To assess the role of surface curvature on the organization of tissues formed by preosteoblast cell cultures, we seeded MC3T3-E1 cells on two types of negative Gaussian curvature surfaces and investigated the development of tissue as a function of time. We used rotationally symmetric capillary bridges (33, 47) as one surface type and compared growth results on these surfaces with tissue growth within pores that are made by casting polydimethylsiloxan (PDMS) around a capillary bridge. In this way, we can compare cell behavior on surfaces with identical Gaussian curvature, but with a mean curvature of opposite sign as well as with opposite signs of principal curvatures (see Materials and methods and Fig. S12).

**Fig. 1.**
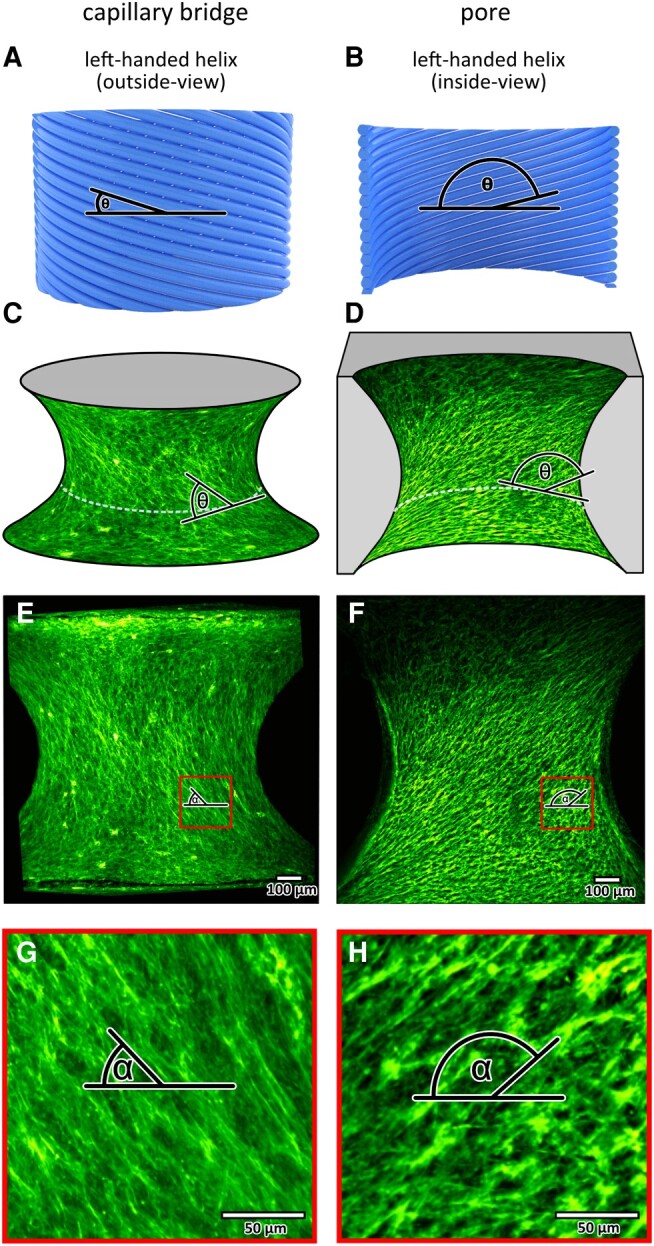
Definition of the fiber angle *θ* based on measurements using a) the outside view or b) the inside view of the same left-handed helix. MC3T3-E1 cells grown on a capillary bridge (c, e, g) and in an oppositely shaped pore (d, f, h) for 32 days and stained for actin. a, d) 3D illustration of the grown tissue (PDMS scaffold shown in gray), the angle *θ* is measured in the 3D surface (0°: horizontal). e, f) MIPs of the actin signal, indicating the fiber angle *α*. g, h) zoom-in into the respective red indicated areas. Note that *α* denotes the fiber angle on the maximum projection image, while *θ* is the fiber angle on the curved surface (see Fig. S13). The two datasets are representative examples.

Before describing the orientation of tissues on these two types of surfaces, it is useful to define clearly what we mean by tissue orientation and how it changes in subsequent tissue layers during growth. This is important as the growth directions of both surfaces go in opposite directions with respect to the rotation axis, making descriptions of chirality and its change with growth sometimes confusing. For our study, we will define growth on the capillary bridges to be outwards and growth on the pore surfaces to be inwards. In a previous study (33), we observed that helical actin stress fibers form in tissues grown on capillary bridges, and we define the helicity of these stress fibers in the same way, with left-handed or right-handed chirality of the helical structures observed. The difficulty is that when we make images of the growing tissues on the two surface types, we do this from different directions with respect to the helical tissue (Fig. 1a and b). For example, on capillary bridges, a left-handed helical tissue, when observed from outside the bridge, would have an angle *θ* lying between 0° and 90° with respect to the negative X-axis of the image. For a tissue with the same helicity but growing within a pore, and observed from the inside, the angle *θ* would lie between 90° and 180° with respect to the negative X-axis of the image (Fig. 1a–d).

We first focus on the quantitative analysis of the actin fiber alignment on a capillary bridge (Fig. 1a, c, e, g) and a pore (Fig. 1b, d, f, h) after 32 days of culturing, which reveals the formation of helical stress fibers with a left-handed chirality for both sample types. The capillary bridges have fiber angles *θ* smaller than 90° (Fig. 1c) and the pores have fiber angles *θ* higher than 90° (Fig. 1d). The different angles measured for the two sample types are both indicating a left-handed chiral pattern. This is due to the differences in imaging conditions: The tissue grown on the capillary bridge is observed from the outside of the helix, and the tissue grown in the pore is observed from the inside of the helix (see Fig. 1a–d). This formation of a tissue with left-handed chirality after 32 days is consistent with previous reports on experiments performed on capillary bridges (33).

### Tissue twisting during growth

The observation described above raises several questions: (i) How consistent is the tendency toward the observed chirality? (ii) At which time point does the chiral pattern emerge and is the actin fiber alignment different in earlier culturing stages? (iii) What is the main factor determining final and potentially transient actin fiber orientations? To address these questions, we chose a time-series approach: Capillary bridges and pore samples were fixed at different time points of culturing (days 4, 7, 11, 16, 23, and 32) and imaged with light sheet microscopy using a fluorescent actin stain.

**Fig. 2.**
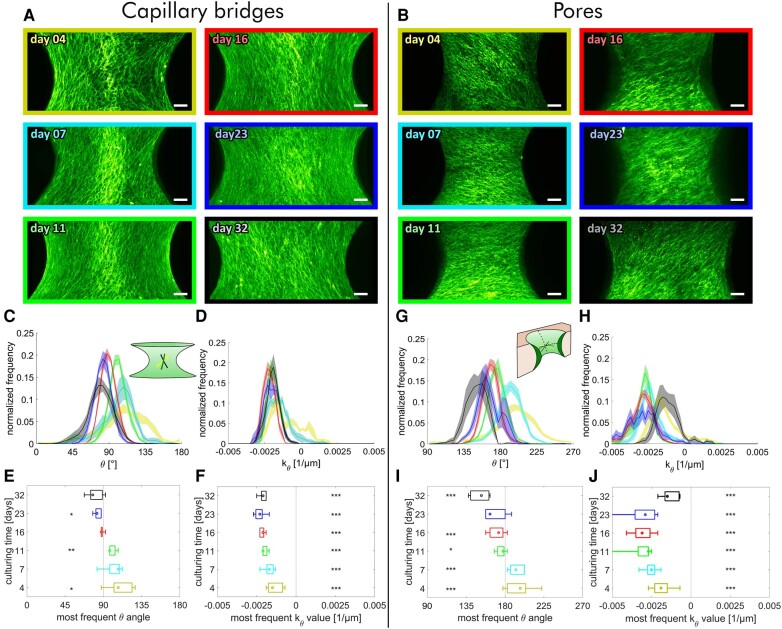
Evolution of the cell orientation of tissues grown a) on capillary bridges and b) in scaffold pores. Images are MIPs of samples stained for actin fixed at day 4 (yellow), 7 (turquois), 11 (green), 16 (red), 23 (blue), and 32 (black), respectively (scale bars: 100 µm). c, g) histograms of the actin fiber angle distribution averaged over all samples with the same culturing time; d, h) histograms of the measured normal curvature along the *θ* direction (*k_θ_*) averaged over all samples with the same culturing time. Shaded regions refer to the SE of the mean for each bin; (e, f, i, j) median, interquartile intervals, and range of the peak *θ* angles, and *k_θ_* values are presented as a box and whisker plot. Single sample histograms of all samples are shown in Figs. S1 and S2. The number of independent samples for every time point are: *n*_CB day 4_ = 5, *n*_CB day 7_ = 5, *n*_CB day 11_ = 5, *n*_CB day 16_ = 5, *n*_CB day 23_ = 5, *n*_CB day 32_ = 5, g–j) *n*_Pore day 4_ = 29, *n*_Pore day 7_ = 17, *n*_Pore day 11_ = 17, *n*_Pore day 16_ = 16, *n*_Pore day 23_ = 7, *n*_Pore day 32_ = 7. Statistical significance between most frequent *θ* angles and 90° (e) or 180° (i) or most frequent curvature *k_θ_* and *k_θ_*  *=* 0.1/µm (f, j) is indicated with *P* < 0.05 (*), *P* < 0.005 (**), and *P* < 0.001 (***).

For capillary bridges, we observed fiber angles *θ* distributed around 90°, meaning stress fibers are oriented close to meridional directions (i.e. fibers are close to being parallel with the pore or bridge rotation axis). At day 4, the tissue orientation (*θ* angle) is right handed and mainly adopts angles higher than 90° ($\mathrm{day}\;\;4:\;\;\bar{\;\theta }=\;110.5^{\circ }$). With ongoing culturing time, $\;\bar{\;\theta }$ gradually decreases and adopts values below 90° after more than 16 days of culturing. This indicates the tissue gains a left-handed chirality during growth (day 32: $\;\bar{\;\theta }=\;80.5^{\circ }$; see Fig. 2c and e).

In contrast to tissue growing on capillary bridges, tissue grown in pores align consistently around the equatorial direction with angles being centered around *θ* = 180° (or 0°). However, in a similar manner to capillary bridge tissues, at early stages (e.g. day 4) the tissue has a right-handed chirality ($\mathrm{day}\;4:\;\;\bar{\;\theta }=\;34.9^{\circ }$) and also gradually shifts toward a left-handed chirality with increasing culturing time (day 32: $\;\bar{\;\theta }=\;151.1^{\circ }\;$ see Fig. 2g and i).

Negative Gaussian curvature implies that any line on a surface, and thus the cells and stress fibers, can have positive, negative, or even zero curvatures, depending on their local orientations. As the sign of curvature is somewhat arbitrary, we define a concave direction as one that has a negative normal curvature in that direction. An example would be the meridional direction on an inward waisting capillary bridge. A convex direction would have positive normal curvature, such as the equatorial line around the waist of a capillary bridge. Clearly, on the pores, the signs of principle curvature swap with respect to the capillary bridges, meaning the equatorial lines are concave and meridians are convex using this definition.

We used 3D models of the experimental surfaces to evaluate the normal curvature $k_{\theta }$ of the fibers. $k_{\theta }$ was found to be relatively constant for the capillary bridges over the entire culturing time with typical values of −0.002 µm^−1^ (Fig. 2d and f), indicating the tissue's tendency to consistently align along slightly negative curvature (concave) directions. Surprisingly, in tissues grown in pores, an alignment along concave directions was also observed (Fig. 2h and j). The curvature distributions of fibers within the pores are broader than those of the capillary bridges. An interesting similarity is that for both sample types, the curvature at day 4 is similar ($\bar{k_{\theta }(\mathrm{pore},\;\mathrm{day}\;4)}=\;-0.0018\;\mu m^{-1}$, $\bar{k_{\theta }(\mathrm{CB},\;\mathrm{day}\;4)}=\;-0.0013\;\mu m^{\text{-}1}$). On both surface types, the normal curvature $k_{\theta }$ becomes more and more negative during growth, with only the last data points of the tissues grown in pores (day 32), showing a slight increase in curvature. For all samples and time points, $k_{\theta }$ was highly significantly different from 0.

The θ values reflect the actin alignment on the tissue surface at different time points and allow us to hypothesize about changes in tissue orientation with growth. In both sample types, the actin fiber-orientation changes from a right-handed helix to a left-handed one during growth. This implies a negative twist for both tissues grown in pores and on capillary bridges. This consistent twisting of the helical orientation of the actin stress fibers as a function of growth time potentially suggests that multiple cell layers form a twisted plywood structure. An alternative explanation is that cells in the bulk reorient themselves with time and, hence, no correlation between fiber orientation on the surface and in the bulk should be found. To test this, we performed an analysis of the fiber orientation as a function of depth of the tissues.

### Conserved fiber orientation of the tissue

**Fig. 3.**
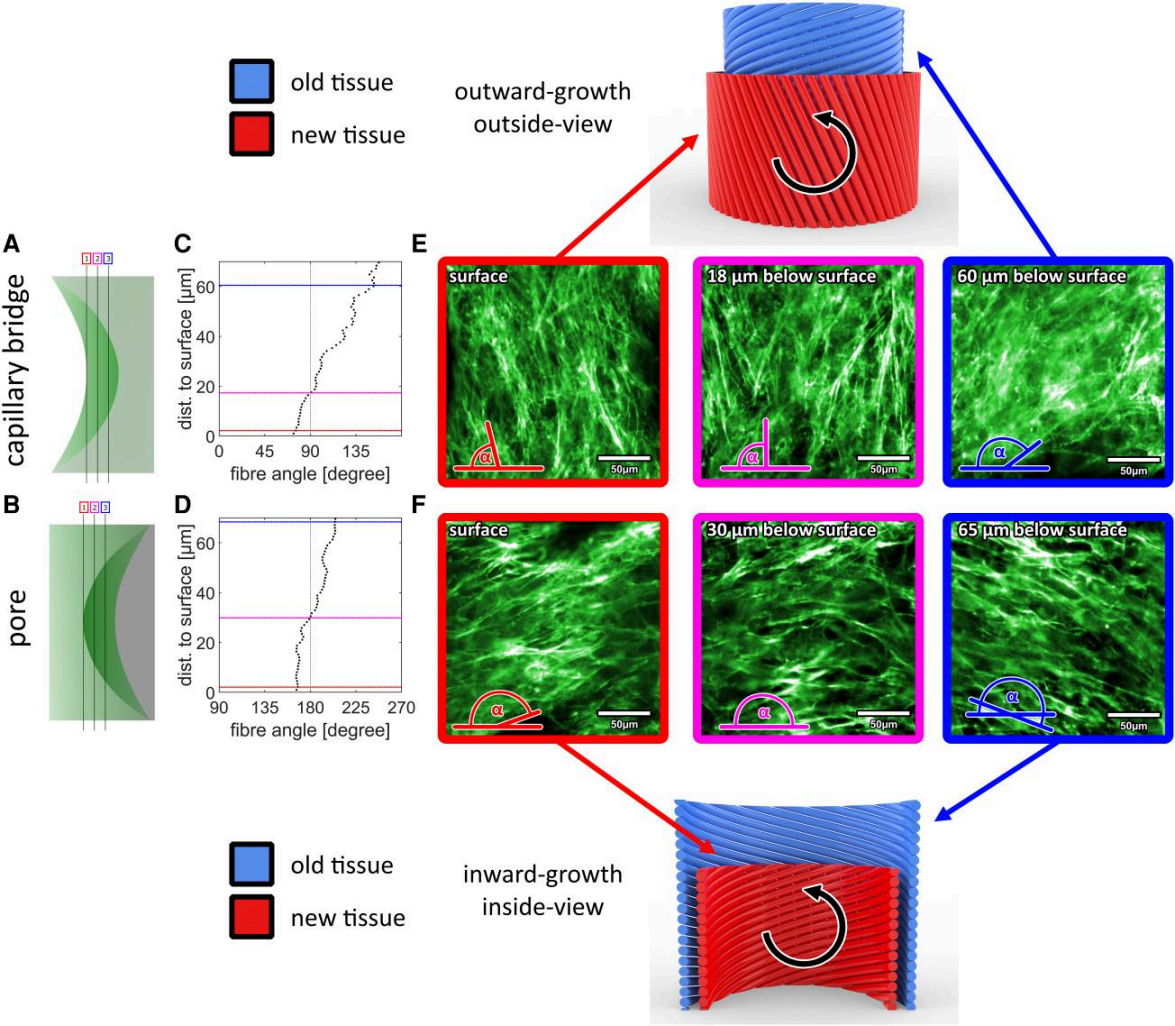
Actin fiber orientations at different depths below the tissue surface of MC3T3-E1 cells grown for 16 days on a capillary bridge (a, c) or inside a pore scaffold (b, d). Each data point represents the result of a fiber-orientation measurement of a single slice within a region of 200 × 200 µm^2^. e, f) Fluorescence images showing the actin fiber arrangement in selected slices (frame color corresponds to line in plot, brightness/contrast is adjusted to compensate for signal loss below the surface). The sketches illustrate the spatial and temporal development of the fiber orientations for both setups. The black arrows in counter-clockwise direction indicate that in both cases a negative twist is present with ongoing tissue growth. The two datasets are representative examples; results for other samples can be found in Figs. S3 and S4 and Videos S1 and S2. Also, video of a depth profile is shown in Video S3.

As the results in Fig. 2 indicate that orientation of newly deposited tissue changes with growth, we next determined how the tissue is organized below the surface. We define any change in helicity with growth as the twist, with a positive twist meaning the angle *θ* increases as the tissue grows (outwards [capillary bridge] or inwards [pore]) and a negative twist means the angle *θ* decreases with increasing layer thickness (Fig. 3, black arrows). To do this, we measured the actin fiber orientation at the neck region of each image of the image stacks derived from the light sheet datasets after 16 days of culturing (see Fig. 3). This time point was chosen as this is the first time point where a flip toward a left-handed chirality is shown (see Fig. 2). On the capillary bridges, the tissue below the surface (∼60 µm) aligns in a right-handed chiral pattern with fiber angles above 90°, whereas the surface tissue exhibits angles below 90° (Fig. 3e). This is consistent with the negative twist observed in surface actin orientation from the time series experiments described above. As also visible in Video S3 (left panel), only one twist event is observed, and there is no clear separation between the cell layers. A similar twisted-plywood-like organization is also observed in the pore samples (Fig. 3f). The two datasets shown in Fig. 3 are representative examples. For both sample types, nearly all of the obtained datasets showed a comparable behavior (Figs. S3 and S4).

### Collagen fibers follow actin stress-fiber orientation

**Fig. 4.**
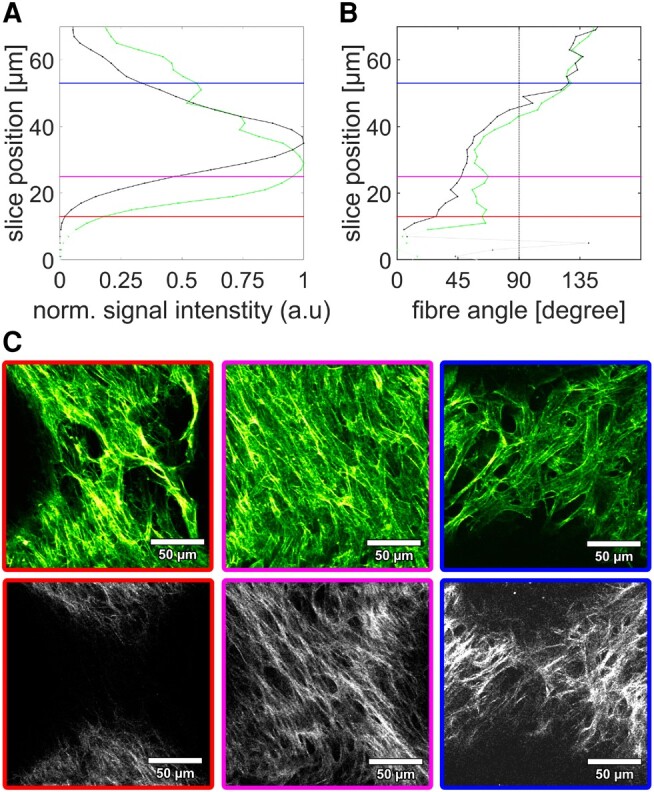
Co-alignment of actin and collagen fibers. a) intensity profiles of the actin (green) and collagen (black) image stacks (moving from outside the sample (0) 70 µm deep into the sample); b) results of the fiber angle evaluation for actin (green) and collagen (black); very low intensity regions are shown in light colors; vertical lines correspond to the selected image slices shown in c): actin (upper panel) and collagen (second harmonic generation signal, lower panel). Image brightness and contrast settings were adjusted for visibility. More datasets are given in Fig. S5 and Video S3.

As MC3T3-E1 cells treated with ascorbic acid (included in our growth medium) are known to synthesize highly ordered collagen fibrils (48), we next investigated the collagen fiber alignment inside the tissue. Confocal and second harmonic generation depth scans were performed to obtain the orientation of collagen and actin fibers of tissue grown on capillary bridges for 16 days. This analysis led to two main results. (i) In the outer layers of the tissue actin is present but no collagen (Fig. 4c, red box), while deeper regions contain both actin and collagen (Fig. 4c, magenta and blue). The offset of the collagen signal relative to the actin is 5–10 µm (Fig. 4a). (ii) From Fig. 4b as well as from the selected slices shown in Fig. 4c, it is clearly visible that actin and collagen fibers are oriented in similar directions. This is observed throughout the entire sample, thus indicating a consistent co-alignment of cellular and extracellular components (see also Videos S1–S3).

### Investigating the role of nuclear mechanosensing, cytoskeleton, and contractility

**Fig. 5.**
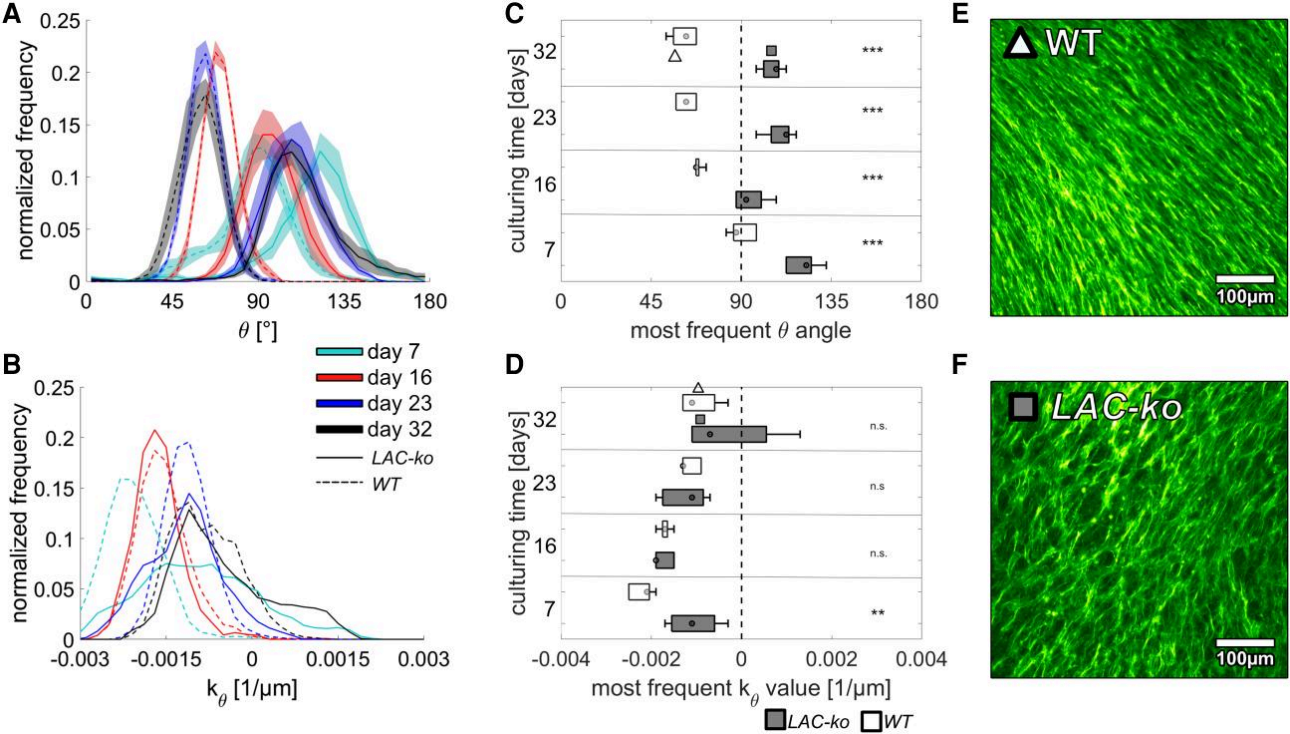
Cell orientations of lamin A/C-deficient MC3T3-E1 cells (LAC-ko, solid lines, gray boxes) compared with WT (dashed lines, empty boxes) grown on capillary bridges. a) Histograms of the actin fiber angle distribution averaged over all samples with the same culturing time. Shaded regions refer to the SE of the mean for each bin. b) Histograms of the measured normal curvatures along the fiber direction averaged over all samples with the same culturing time. c) Mean and standard distribution of the peak *θ* angles and d) the curvature along the fiber direction. e, f) Two examples of the center regions after 32 days of culturing. Symbols are used to indicate the respective values in c) and d). Sample numbers: *n* = 5 for each group. Histograms for the single samples are found in Fig. S7. Statistical significance between WT and LAC-ko for each time point is indicated with not significance (n.s.), *P* < 0.005 (**) and *P* < 0.001 (***).

To obtain mechanistic insight into the change of the cellular orientation during culture, we first tested the impact of lamin A/C deficiency associated with impaired mechanosensing of the nucleus (49). Therefore, lamin A/C-deficient MC3T3-E1 cells (LAC-ko) were generated using the CRISPR/Cas9 system (for details, see supplementary material and Fig. S6) and were grown on capillary bridge samples. At the beginning of cell culture (day 7), LAC-ko cells show a right-handed chirality with *θ* angles clearly larger than observed in WT cells. In contrast to the WT cells, there was no twist of the growing tissue toward a left-handed chirality (see Fig. 5). Instead, the tissue retained the original right-handed chiral pattern even after 32 days of tissue culture. The development of *k_θ_* was remarkably similar in both groups (Fig. 5d). Visually, the tissue formed by the LAC-ko cells appears to be much looser compared with the WT tissue (Fig. 5e and f).

To investigate the effect of the cytoskeleton and contractility, MC3T3-E1 cells grown on capillary bridges were treated with either Blebbistatin (inhibition of myosin II), TGF-β1 (stabilization of actin stress fibers; decreased motility), or Latrunculin A (LatA; inhibition of actin fiber assembly). Samples were fixed on days 7 and 32. Blebbistatin-treated samples show at days 7 and 32 a left-chiral alignment, while the controls exhibit a flip from right to left during this period (Fig. S8). In contrast, TGF-β1 treatment leads to right chiral arrangement at both time points, indicating a conservation of the right handedness (Fig. S9). With LatA treatment, a nonsignificant trend toward smaller *θ* angles was observed for day 7, while *θ* was significantly smaller compared with control at day 32, where also a left-handed chirality was observed as in the controls (Fig. S10).

To exclude the effect of dimethyl sulfoxide (DMSO), which is used as a solvent for Blebbistatin and LatA, additional samples were treated with DMSO only. In this case, no significant effect on the tissue organization compared with the control samples was observed (Fig. S11).

## Discussion

There are three main aspects that we learn from observing the time- and depth-dependent organization of tissues formed by preosteoblast (MC3T3-E1) cells on negative Gaussian curvature surfaces:

- We observed that during cell culture, there was a consistent change in the orientation of the surface tissue layer relative to the tissue directly beneath it. This suggests that the cells sense underlying tissue orientation and then produce a new orientation with a negative twist. Depth profile measurements demonstrated that this arrangement is preserved in the bulk, thus resulting in tissue organized similar to parts of a “twisted-plywood-like tissue” (1). Our results further suggest that the cells located below the surface layer (∼10 µm below the tissue surface) lay down a collagen matrix which is co-aligned with the cell's actin cytoskeleton, thus hinting at the mechanism giving rise to extracellular matrix organization in such tissues.
- The normal curvature along the main actin fiber direction was always negative (concave). Despite the change in mean surface curvature during growth, the normal curvature remained close to −0.002 µm^−1^ for both, capillary bridges and pores (with a larger scatter in the data for the latter). This suggests a mechanism by which a twist in orientation appears in order for the growing tissue layer to retain this preferred curvature. A consequence of this is that fiber orientation in a growing tissue is strongly mediated by changes in surface geometry due to growth.
- The results reveal a consistent change in tissue chirality. While in early tissue culture stages, actin stress fibers and collagen are oriented in a right-handed chiral structure (capillary bridges: 90 < θ < 180, pores: 180 < θ < 270), after around 2 weeks of tissue culture a left-handed chirality is adopted. This behavior revealed to be remarkably similar for both sample types. It is still unclear how the first layer of tissue becomes ordered in the early stages of growth and what triggers the observed changes in orientation.

We hypothesize that the underlying mechanism of tissue patterning is linked to the mechosensitivity of the employed cells. Compared with the WT tissues, tissues formed by a lamin A/C-deficient cell line as well as those of tissues treated with drugs to modulate actin–myosin contractility displayed modified twisting behavior. After short culturing times, lamin A/C-deficient cells and cells treated with Blebbistatin or TGF-β1 form spiral patterns, but different to WT cells, longer culturing times do not lead to a change in chirality and thus to the absence of tissue twisting. This might indicate that especially the extent of twisting is sensitive to disturbances in the actin skeleton and nuclear lamins. This hypothesis is supported by observations that Blebbistatin treatments lead to less pronounced stress fibers and a modification in tissue structure (33, 50). Other recent studies have shown a connection between curvature, matrix stiffness, actin cytoskeleton, and lamin A/C levels. For example, lamin A/C levels increase with curvature as well as with surface stiffness, the latter being dependent on actomyosin (51–54). Although the exact mechanism underlying cell layer alignment and twisting is not yet known, it is possible that Yes-associated protein 1 signaling is involved, which functions in mechanotransduction and bone development and is decreased by lamin A/C deficiency (51, 55, 56). Additionally, impaired cell migration, which has been observed in fibroblasts lacking lamin A/C (57), might also be accountable for the defective twisting behavior in our LAC-ko cells. Cell migration plays an essential role in the ordered formation of bone tissue (58) and is influenced by surface topology (26, 59, 60). Another aspect that remains open is the role of the preexisting extracellular matrix on cell alignment. Connections between cells, giving rise to the stress fibers, and extracellular matrix enables geometric sensing to occur at length scales much larger than the size of a single cell itself (36, 37). More detailed imaging will be required to fully understand this.

Low doses of LatA were shown in literature to result in inverted chirality of fibroblasts in single cell and tissue sheets (42). This was not observed in our study on MC3T3-E1 cells. The drug, however, led to a more pronounced clockwise rotation of the tissue which is observed by smaller *θ* angles at the end of the culture compared with the control samples. The inhibiting effect of TGF-β1 on the twist might be explained by a higher contractility of the cells as well as their decreased motility (61). Surprisingly, the decrease of contractility by Blebbistatin led already at the beginning of the tissue formation to a left-handed orientation, which continued to be so until the end of the culture. Although the specific mechanisms remain unknown, these data suggest that the emergence of chirality and tissue twisting is under cellular control and is influenced by nuclear mechanosensing and actin cytoskeleton properties.

As the chosen MC3T3-E1 cells are preosteoblastic, it is remarkable that the observed tissue twisting presents similarities of what is found in bone. Osteons located in human long bone, for example, exhibit a typical lamellar structure which originates from mineralized collagen bundles with periodically changing fiber angles (9–13). While in our *in vitro* setup, a geometrically relatively simple twisting pattern was observed, 3D electron microscopy of human osteons revealed a more sophisticated geometry of the twisting fibers (15). Hence, the results of the *in vitro* approach do not reflect the full complexity of the collagen alignment in bone. Despite the differences in tissue organization and composition (a highly mineralized collagen extracellular matrix vs. a loosely packed cell-collagen tissue), it is likely that the emergence of a chiral tissue structure observed in our *in vitro* model system is triggered by similar processes responsible for the twisted structures seen *in vivo* within bone. This difference in tissue composition also has implications for potential growth mechanisms. Since lamellar bone is formed via appositional growth (osteoblasts laying down a collagen-rich matrix which eventually mineralizes) in the presented study, we found a cell-rich tissue potentially also allowing for interstitial growth via cell proliferation or the secretion of ECM below the tissue surface. Since this work mainly focuses on tissue alignment, the lack of knowledge about the dominant growth mechanism is a limitation and needs to be explored in future experiments. This is especially important when investigating mechanisms leading to the overall tissue shape. We previously demonstrated that tissue grown by the same cell line on capillary bridges adopts shapes known from fluids with isotropic surface tension, thus forming constant-mean curvature (Delaunay) surfaces (33). Further theoretical work investigated how the presence of fibrous tensile elements that follow geodesic lines on the surface, may modulate tissue shape, as it might be the case for highly aligned and orientated cell systems (34). For the fiber angles observed in experiments, macroscopic surface geometries were remarkably similar to the Delaunay surfaces. The main difference is that fiber angles can strongly modulate internal tissue pressure, thus strengthening the role of mechanosensing in tissue orientation.

Neville cites three possible mechanisms leading to the formation of helicoidal or twisted plywood tissues in biological materials (1): self-assembly of molecules, directed assembly by cellular mechanisms, or mechanical reorientation. We suggest that in our model system, the development of a twisted, plywood-like tissue relies on a mixture of the first and second mechanisms, in which the cells self-organize into ordered arrays resembling liquid crystals, and then control collagen alignment. Although Neville’s work considered molecules which passively self-assemble also contractile forces mediated by the cell's cytoskeleton may be considered in growing biological tissues (34). Further data suggest that cells may also spontaneously align like liquid crystals on a surface (20, 62, 63). Hence, it remains open if the tissue alignment observed in our experiments is a result of a direct cellular response to the imposed curvature, if physical principles are utilized to create a complex shape or if the observed pattern is formed by simply adapting an energetically preferable state due to the cell's contractile properties. Although our data provide insight into the mechanism for tissue organization, we, however, cannot say why the first layer of cells has the orientation it does as in principle both left- and right-handed chirality of tissue layers should allow cells to experience identical curvatures. Interestingly, there are examples found in nature of inherently unstable inanimate systems, where slight disturbances lead to an emerging chiral-like pattern as found in the plughole vortex in a bath tub. When removing a plug from a tub filled with perfectly still water, theoretically no vortex will be formed. However, even the smallest asymmetry leads to the formation of a complex wrinkled chiral structure (64). As energetically left and right chiral vortices are identical, the chirality of the final structure depends on the initial disturbance. If the cells on the curved surface in our experiment form an unstable system upon confluency, it is thinkable that the intrinsic left-right polarity acts as such a disturbance and, hence, determines the direction of the emerging pattern. Indeed, it was shown that different cell lines, including the cell-line employed in this project, exhibit a left-right polarity (42, 65). Once the first layer of cells has formed, subsequent cell layers are deposited in manner such that they can keep aligned with the preferential curvature direction giving rise to a twist relative to the layer below. As the tissue thickens collagen is produced in an already helical environment of cells and tissues, thus freezing this helical arrangement into place. This is in contrast with the hypothesis that procollagen molecules form a liquid crystal phase and, thus, shape the emerging tissue in bone (66). This alternative hypothesis is based on the observation that also in the absence of cells under well-controlled conditions, collagen can spontaneously form cholesteric liquid crystalline phases similar to a twisted plywood arrangement. Since it remains open to which extent this process may take place in a more complex environment, our results do not dispute the importance of self-organization processes of collagen but show that also processes at the level of cells can give rise to the emergence of twisted helices. It is becoming clearer that cellular control plays a crucial role; however, we still do not understand how cells sense curvature although data give some hints as to the importance of mechanosensing. The hypothesis that the twist in tissue orientation is linked to changing curvature opens up some new predictions, which potentially can be tested experimentally. During remodeling of cortical bone, osteoblasts follow the cutting cone of osteoclasts depositing oriented tissue in the osteoclasts wake. If surface curvature indeed mediates cell and collagen orientation, then the speed of cell migration, and osteoclast resorption would also play an important role in tissue orientation, perhaps explaining the variation in osteonal structures seen *in vivo* (67).

In conclusion, the presented results reveal the importance of surface curvature for tissue alignment in a model system for bone formation. Curvature sensing, mediated by actin–myosin cell contractility and the nuclear lamins, enables the cells to align in particular curvature directions which change as growth proceeds. This change results in the formation of a layer of twisted-plywood-like tissue. We demonstrate that such tissues can be formed *in vitro*, this alone gives us a new model system to help understand how complex multifunctional materials are formed by fibrous building blocks and to potentially control and optimize tissue orientation in the future.

## Materials and methods

### Manufacturing of PDMS capillary bridges

Capillary bridges were fabricated, according to the routines described previously (33, 47). For the fabrication of the scaffolds, the Sylgard 184 Elastomer Kit (Dow Corning, USA) was used with a mixing ratio of 10 parts base with 1 part curing agent (weight:weight). After mixing the base with the curing agent, PDMS was degassed in vacuum for 20 min. Using a syringe pump (New Era Pump Systems, NY, USA), the PDMS was transferred to cylindrical aluminum pillars facing to each other with a radius of 1 mm. The amount of liquid dispensed on the pillars corresponding to the volume of the final capillary bridges and, therefore, was adjusted for the individual capillary bridge sizes. The pillars were moved together so that the PDMS liquid droplets got in contact and a liquid bridge formed between two pillars. The distance between the pillars was adjusted to 1.25 mm, which corresponds to the height of the resulting capillary bridges. The capillary bridges were cured at 120°C for 20 min.

### Manufacturing PDMS pores with negative Gaussian curvature

The capillary bridges manufactured above were furthermore used as templates for pores with a negative Gaussian curvature. A thin layer of PDMS (see mixing procedure in the last paragraph) was applied on a 4-inch silicon wafer by spin coating (1,100 rpm, 1 min, spin coater, Laurell Technologies Corporation, PA, USA). The capillary bridges were placed with a tweezer on the wafer and cured for 2 h at 80 °C. For the casting of PDMS, a method described in Ref. (68) was used: The wafer with the capillary bridges was treated with oxygen plasma (50 W, 1 min, Emitech K1050X) and afterwards directly placed in a bath of 100% ethanol for 30 min in vacuum and dried for 30 min at 80°C. A polymethylmethacrylat frame was glued on the wafer surrounding the capillary bridges and filled with PDMS while avoiding complete coverage of the capillary brides. Finally, the samples were placed in vacuum for 20 min and cured at 80°C for 2 h. The PDMS with the pores was manually detached from the capillary bridges and cut into an appropriate size for cell culture. A graphical description is shown in Fig. S12.

### Functionalization of PDMS scaffolds

The surfaces of the PDMS scaffolds were functionalized using a combination of plasma treatment, (3-aminopropyl)triethoxysilane (Sigma-Aldrich Chemie GmbH, Steinheim, Germany) and glutaraldehyde (Carl Roth GmbH + Co, Karlsruhe, Germany) to covalently bind fibronectin (Sigma-Aldrich Chemie GmbH) to the surface. The method was adapted from Tan and Desai (69) and described in detail in Ehrig et al. (33).

### Cell line

For all experiments, except the lamin A/C deficient and the collagen imaging, MC3T3-E1 cells were used. For the lamin A/C-deficient experiments, MC3T3-E1 LAC-ko and MC3T3-E1 cells were provided by the Ludwig Boltzmann Institute of Osteology. MC3T3-E1 cells were also used for imaging the collagen organization. LAC-ko cells were generated using the CRISPR/Cas9 system (for details, see supplementary material and Fig. S6).

All cells were tested regularly for mycoplasma contamination, and the MC3T3-E1 cells were authenticated by short tandem repeat (STR) analysis (Microsynth, Balgach, Switzerland).

### Cell culture

Murine preosteoblast cells MC3T3-E1 were seeded on the scaffolds with a density of 10^5^ cells/cm² and cultured using α-minimum essential medium (Sigma-Aldrich Chemie GmbH) supplemented with 4,500 mg/L D-(+)-glucose (Sigma-Aldrich Chemie GmbH), 10% fetal bovine serum (Gibco, Life Technologies Limited, Paisley, UK), 50 µg/mL L-ascorbic acid (Sigma-Aldrich Chemie GmbH), and 1% penicillin–streptomycin (Sigma-Aldrich Chemie GmbH). The samples were incubated at 37°C and 5% CO_2_ in humidified atmosphere. Cell seeding resulted in mainly separated cells which form a confluent layer after typically 2–3 days of culturing. The scaffolds were transferred into new plates every 7 days starting from day 4. The cell culture media was exchanged every 2 to 3 days beginning at day 4 unless otherwise stated.

### Blebbistatin/TGF-β1/Latrunculin A

To change the contractility of the cytoskeleton, the treatment of the samples with three different drugs was performed starting at day 4 after seeding until the end of the experiment: myosin II inhibitor Blebbistatin to inhibit the myosin–actin contractility; TGF-β1 to enhance the contractility and stabilize the cytoskeleton or Latrunculin A to inhibit actin assembly. (−)-Blebbistatin (Sigma-Aldrich Chemie GmbH) was added to the growth media at a final concentration of 2 µM with 0.1% DMSO (Sigma-Aldrich Chemie GmbH). The recombinant human TGF-β1 (Invitrogen, MD, USA) was added at a final concentration of 1 ng/mL. The actin monomer-binding toxin Latrunculin A (Millipore, Darmstadt, Germany) was added at a final concentration of 20 nM with 0.1% DMSO. To exclude the effect of the solvent DMSO, additional samples were treated with 0.1% DMSO in the same period.

### Fluorescent staining and imaging

The tissue was fixed at the end of each experiment with 4% paraformaldehyde in phosphate-buffered saline (PBS; Alfa Aesar, ThermoFisher GmbH, Kandel, Germany) for 5 min at room temperature followed by thoroughly washing using 1× Dulbecco’s PBS (DPBS; Sigma-Aldrich Chemie GmbH). The tissue was permeabilized with 1% Triton X-100 (Sigma-Aldrich Chemie GmbH) between 3 h to overnight at 4°C and washed extensively with 1× DPBS. To visualize F-actin, samples were stained for 90 min with 1.65 × 10^−7^ M Alexa Fluor 488 phalloidin (Invitrogen, Life Technologies Corporation, Oregon, USA) in 1× DPBS. The tissue was again washed with 1× DPBS and to visualize the cell nuclei, the tissue was incubated for 5 min with 1 µM TO-PRO-3 iodide (Invitrogen, Life Technologies Corporation) followed by washing with 1 × DPBS (nuclei data not shown). Prior to the imaging of the pore samples, after fixation and staining, the samples were cryo-embedded in optimal cutting temperature compound embedding matrix (Cellpath, Newtown, UK) and opened by cutting into half, followed by embedding 1% low melting agarose (Carl Roth GmbH + Co) for imaging at room temperature. For fluorescence imaging of capillary bridges and opened pores, a Zeiss Z1 light sheet fluorescence microscope was used. The fixed tissue was placed in the imaging chamber filled with deionized H_2_O. A Plan Apochromat 20×/1.0 Corr DIC water immersion objective lens was used and a 488- and 633-nm excitation laser. To image F-actin, the SBS LP560 beam splitter was employed. 3D data were generated by performing z-stacks with 0.63 µm lateral pixel size and 1.2 µm z-step size. For further analysis, maximum intensity projections (MIP) of the stacks were used. Due to the attenuation of the fluorescence signal from regions inside the tissue, actin fibers on the surface are distinctly brighter than those below. Hence, in the MIPs, mainly the tissue surface is visible. For imaging collagen with two-photon excitation, a SP8 (Leica Microsystems GmbH, Wetzlar, Germany) confocal laser-scanning microscope equipped with pulsed tunable laser (Maitai, Newport Corporation, CA, USA) was used. The excitation wavelength was set to 910 nm while acquiring the emission signal in a spectral window between 430 and 470 nm. The Alexa Fluor 488 phalloidin signal was also acquired for each slice. Image stacks were obtained with a voxel size of 0.606 × 0.606 × 2 µm. The images were acquired using a Fluostar VISIR 25x/0.95 water objective.

### Analysis of fiber alignment

The actin fiber alignment was derived from light sheet microscopy fluorescence data of fixed and stained samples. Due to the anisotropic resolution, instead of using full 3D data, our results are based on MIPs as described above. In short: First, MIPs of the fluorescence images were performed, obvious artifacts, like dust particles, were masked and excluded from the further analysis, and the fiber direction in the projection plane was derived (*α*). To calculate the fiber angle on the sample surface (θ), vectors representing the fiber orientation were projected on rotational symmetric representations of the scaffold using in-house developed Matlab routines (Mathworks Inc., MA, USA, v 2020a). Edge regions as well as regions more than 300 µm above/below the neck center were excluded from the evaluation to avoid boundary artifacts. The routines for the fiber angle alignment are based on the work published in Ref. (33). In ∼80% of the capillary bridge samples and ∼20% of the pore samples, it was possible to also obtain *θ* angles from the backside or second sample half, respectively. In this case, data from both measurements are included in the respective histograms. For every location where *θ* was evaluated, also $\;k_{\theta }$, the normal curvature in *θ* direction was derived. This was done by first calculating the principal curvatures (largest and smallest curvature) at the location and then using the Euler theorem of differential geometry to derive the curvature in the direction of θ. A more detailed description of the evaluation procedure is summarized in the supplementary material and Fig. S13.

### Statistical analysis

Statistical analyses were performed using Matlab. To compare two sample cohorts, the most frequent *θ* or $k_{\theta }$ value of every sample (histogram peak position) served as input data for a two sample t test. When testing whether *θ* or $k_{\theta }$ are significantly different to a given value, a one sample t test was chosen. Differences of *P* < 0.05 are considered as significant.

## Supplementary Material

pgae121_Supplementary_Data

## Data Availability

Images and scripts used for the fiber analysis are available via the open Zenodo data repository https://zenodo.org/ (doi: https://doi.org/10.5281/zenodo.10816679). In addition to the MIPs, also two examples of full 3D datasets are provided (one capillary bridge and one pore). All full 3D raw datasets are stored at the local server of the University of Salzburg and are available to interested scientists upon request.

## Preprints

An earlier version of this manuscript was posted on a preprint server: https://doi.org/10.1101/2023.09.04.556075.
